# Postoperative liver dysfunction is associated with poor long-term outcomes in patients with colorectal cancer: a retrospective cohort study

**DOI:** 10.1186/s12876-023-02762-y

**Published:** 2023-04-18

**Authors:** Shutaro Sumiyoshi, Jun Kiuchi, Yoshiaki Kuriu, Tomohiro Arita, Hiroki Shimizu, Wataru Takaki, Takuma Ohashi, Yusuke Yamamoto, Hirotaka Konishi, Ryo Morimura, Atsushi Shiozaki, Hisashi Ikoma, Takeshi Kubota, Hitoshi Fujiwara, Kazuma Okamoto, Eigo Otsuji

**Affiliations:** Division of Digestive Surgery, Department of Surgery, Kyoto Prefectural University of Medicine, 465 Kajii-Cho, Kyoto, Kawaramachihirokoji, Kamigyo-Ku Japan

**Keywords:** Colorectal cancer, Postoperative complication, Liver dysfunction, Prognosis, Risk factor, Fatty liver

## Abstract

**Background:**

Postoperative hepatobiliary enzyme abnormalities often present as postoperative liver dysfunction in patients with colorectal cancer. This study aimed to clarify the risk factors of postoperative liver dysfunction and its prognostic impact following colorectal cancer surgery.

**Methods:**

We retrospectively analyzed data from 360 consecutive patients who underwent radical resection for Stage I–IV colorectal cancer between 2015 and 2019. A subset of 249 patients with Stage III colorectal cancer were examined to assess the prognostic impact of liver dysfunction.

**Results:**

Forty-eight (13.3%) colorectal cancer patients (Stages I–IV) developed postoperative liver dysfunction (Common Terminology Criteria for Adverse Events version 5.0 CTCAE v5.0 ≥ Grade 2). Univariate and multivariate analyses identified the liver-to-spleen ratio on preoperative plain computed tomography (L/S ratio; *P* = 0.002, Odds ratio 2.66) as an independent risk factor for liver dysfunction. Patients with postoperative liver dysfunction showed significantly poorer disease-free survival than patients without liver dysfunction (*P* < 0.001). Univariate and multivariate analyses using Cox’s proportional hazards model revealed that postoperative liver dysfunction independently was a poor prognostic factor (*P* = 0.001, Hazard ratio 2.75, 95% CI: 1.54–4.73).

**Conclusions:**

Postoperative liver dysfunction was associated with poor long-term outcomes in patients with Stage III colorectal cancer. A low liver-to-spleen ratio on preoperative plain computed tomography images was an independent risk factor of postoperative liver dysfunction.

**Supplementary Information:**

The online version contains supplementary material available at 10.1186/s12876-023-02762-y.

## Background

Colorectal cancer (CRC) is the third most commonly occurring cancer in the world [1]. Despite recent advances in surgical procedures and chemotherapy, tumor recurrence is often observed in CRC patients after curative resection, leading to a high mortality rate. Therefore, it is important to identify reliable predictors of patients at high risk of tumor recurrence.

The association between postoperative adverse events and poor long-term outcomes has been reported in various surgical fields [2–4]. In CRC, postoperative complications were associated with tumor recurrence and poor outcomes [5]. Therefore, postoperative liver dysfunction may also be associated with the deterioration of long-term outcomes. Although postoperative hepatobiliary enzyme abnormalities often present as postoperative liver dysfunction in patients with CRC, there have been no reports concerning the prognostic impact of postoperative liver dysfunction, to the best of our knowledge.

In this study, we retrospectively identified putative risk factors for the occurrence of postoperative liver dysfunction and investigated the prognostic impact of postoperative liver dysfunction among CRC patients who received radical resection. Our results will provide evidence to clarify the association between postoperative liver dysfunction occurrence and poor prognosis in patients with CRC.

## Methods

### Patients

**Fig. 1:**
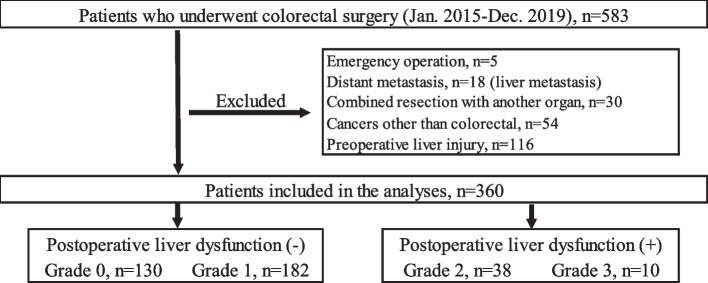
Flowchart for selecting and classifying subject

This study was conducted in accordance with the ethical standards of the Kyoto Prefectural University of Medicine and the Declaration of Helsinki. Written informed consent was obtained from all patients. The experimental protocol was approved by the Ethical Review Board of the Kyoto Prefectural University of Medicine (ERB-C-1178–1). We retrospectively analyzed data from 583 consecutive patients who underwent surgical resection with lymph node dissection for Stages I–IV colorectal cancer between 2015 and 2019. Patients were excluded if they had preoperative hepatobiliary enzyme abnormalities (Common Terminology Criteria for Adverse Events version 5.0 CTCAE v5.0 ≥ Grade 1), distant metastasis including liver metastasis, other cancers, received colectomy with combined resection, or had undergone an emergency operation. All 18 cases of distant metastasis were liver metastasis, with no metastasis to other organs. Ultimately, 360 patients were included in this study. Patient characteristics, pathological and surgical findings, and postoperative clinical courses were reviewed from medical records and databases at our institution (Fig. 1). In addition, to assess the prognostic effect of postoperative liver dysfunction, we retrospectively analyzed data from 249 consecutive patients who underwent radical resection for Stage III colorectal cancer.

The preoperative diagnosis of colorectal adenocarcinoma was confirmed by endoscopy and biopsy. Patients with lower rectal cancer, clinically diagnosed as T3 or deeper, underwent neoadjuvant chemoradiotherapies followed by radical surgery. The patients underwent colorectal resection and lymph node dissection according to the guidelines of the Japanese Society for Cancer of the Colon and Rectum [6]. Surgical oncologists agreed on all surgical procedures in a preoperative multidisciplinary conference. Tumor stages were determined based on the International Union Against Cancer tumor, node, and metastasis classification system (8^th^ ed.) [7]. Macroscopic and histological cancer types were classified according to the Japanese Classification of Colorectal Carcinoma (8^th^ ed.) [8]. Physical examinations, blood tests, including tumor markers such as carcinoembryonic antigen (CEA), computed tomography (CT), and colonoscopies, were conducted every 3–6 months after surgery. Tumor recurrence was confirmed radiographically, and treatments were initiated immediately.

A range of clinical factors were examined for their association with postoperative liver dysfunction in CRC patients. Serum levels of aspartate transaminase (AST), alanine aminotransferase (ALT), alkaline phosphatase (ALP), gamma-glutamyl transpeptidase (GGT), and total bilirubin (T-BIL) were evaluated in all patients. Hepatobiliary enzyme abnormalities were assessed using CTCAE v5.0 diagnostic criteria as recommended by the Council for International Organizations of Medical Sciences (Supplementary Table 1). Serum hepatobiliary enzyme levels were measured routinely preoperatively, on postoperative days 1, 3, 5, and 7, and based on the postoperative course thereafter. In addition, serum liver enzyme levels were measured at every outpatient visit. Postoperative liver dysfunction was defined by postoperative hepatobiliary enzyme abnormalities of Grade 2 or higher, while severe postoperative liver dysfunction was defined by hepatobiliary enzyme abnormalities of Grade 3 or higher.

### Risk factor identification and prognostic impact

**Fig. 2:**
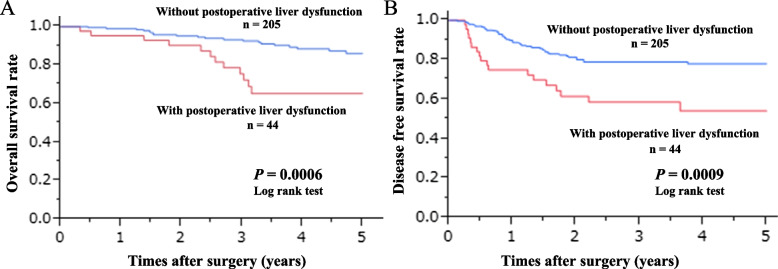
Overall survival curves (A) and disease-free survival curves (B) of Stage III CRC patients with and without postoperative liver dysfunction

First, we investigated potential clinicopathological factors to identify the independent risk factors of postoperative liver dysfunction using univariate and multivariate logistic analyses (Table 1). Second, we examined the putative risk factors for severe postoperative liver dysfunction (Table 2). Then, we observed the features of liver dysfunction during the postoperative period (Table 3). Finally, to confirm the clinical effects of postoperative liver dysfunction, we compared the survival curves of patients with or without postoperative liver dysfunction following radical resection for stage III colorectal cancer (Fig. 2). The prognostic effects of postoperative liver dysfunction were investigated using univariate and multivariate analyses with Cox’s proportional hazard ratios (Table 4).

**Table 1:** Univariate and multivariate analyses of the potential risk factors for postoperative liver dysfunction

Variables	Postoperative liver dysfunction				Univariate^a^	Multivarate^b^		
	(** +**)	( ***n*** = **48**)	(**-**)	( ***n*** =** 312**)	***p***-**value**	**OR** ^c^	**95%CI** ^d^	***p***-**value**
Gender								
Female	20	(13%)	138	(87%)	0.682			
Male	28	(14%)	174	(86%)				
Age (years)								
< 65	18	(16%)	96	(84%)	0.356			
≥ 65	30	(12%)	216	(88%)				
Body composition								
< BMI 25	37	(13%)	250	(87%)	0.629			
≥ BMI 25	11	(15%)	62	(85%)				
Drinking								
No	18	(10%)	163	(90%)	0.056			
Yes	30	(17%)	149	(83%)				
HBs-Ag								
negative	48	(13%)	310	(87%)	0.523			
positive	0	(0%)	2	(100%)				
HCV-Ab								
negative	48	(11%)	307	(89%)	0.275			
positive	0	(0%)	5	(100%)				
Low-molecular-weight heparin								
No	0	(0%)	18	(100%)	**0.031**			
Yes	48	(14%)	294	(86%)				
Neoadjuvant chemotherapy								
No	37	(12%)	282	(88%)	0.258			
Yes	11	(27%)	30	(73%)				
L/S ratio								
≥ L/S 1.268	16	(8%)	183	(92%)	**0.001**	1		
≤ L/S 1.268	32	(20%)	129	(80%)		2.66	1.42–5.18	**0.002**
ALBI score								
≤-2.6	35	(16%)	183	(84%)	0.108			
> -2.6	13	(9%)	129	(91%)				
FIB-4 index								
< 1.3	21	(13%)	138	(87%)	0.928			
≥1.3	27	(13%)	174	(87%)				
Hepatic stenosis index								
< 30	45	(13%)	290	(87%)	0.712			
≥30	3	(12%)	22	(88%)				
Operative time								
< 280 min	29	(12%)	218	(88%)	0.196			
> 280 min	19	(17%)	94	(83%)				
Estimated blood los								
< 50 mL	39	(14%)	249	(86%)	0.815			
≥ 50 mL	9	(13%)	63	(87%)				
Intraoperative blood transfusion								
No	47	(13%)	304	(87%)	0.838			
Yes	1	(11%)	8	(89%)				
Surgical site infection								
No	44	(13%)	287	(87%)	0.949			
Yes	4	(14%)	25	(86%)				
Postoperative ileus								
No	47	(14%)	299	(86%)	0.457			
Yes	1	(7%)	13	(93%)				
Anastomotic leakage								
No	45	(13%)	305	(87%)	0.112			
Yes	3	(30%)	7	(70%)

**Table 2:** The clinical factors of patients with severe (CTCAE Grade ≥ 3) and non-severe (CTCAE Grade 2) postoperative liver dysfunction

Variables	CTCAE classification				Univariate^a^
	Grade 2	(n=38)	Grade 3	(*n*=10)	*p*-value
Gender					
Female	18	(93%)	2	(7%)	0.077
Male	20	(68%)	8	(32%)	
Age (years)					
< 65	16	(89%)	2	(11%)	0.183
≥ 65	22	(73%)	8	(27%)	
Body composition					
< BMI 25	30	(81%)	7	(19%)	0.558
≥ BMI 25	8	(73%)	3	(27%)	
Drinking					
No	14	(78%)	4	(22%)	0.854
Yes	24	(80%)	6	(20%)	
Low-molecular-weight heparin					
No	0		0		
Yes	38	(79%)	10	(21%)	
Neoadjuvant chemotherapy					
No	34	(79%)	9	(21%)	0.602
Yes	4	(80%)	1	(20%)	
L/S ratio					
≥ 1.268	12	(75%)	4	(25%)	0.619
< L/S 1.268	26	(81%)	6	(19%)	
ALBI score					
< -2.6	29	(88%)	4	(12%)	0.032
> -2.6	9	(60%)	6	(40%)	
FIB-4 index					
< 1.3	18	(86%)	3	(14%)	0.228
≥ 1.3	20	(74%)	7	(26%)	
Hepatic stenosis index					
< 30	36	(79%)	10	(21%)	0.263
≥ 30	2	(100%)	0	(0%)	
Anastomotic leakage					
No	36	(82%)	8	(18%)	0.173
Yes	2	(50%)	2	(50%)	
Surgical site infection					
No	36	(82%)	8	(18%)	0.173
Yes	2	(50%)	2	(50%)	
Postoperative ileus					
No	37	(79%)	10	(21%)	0.507
Yes	1	(100%)	0	(0%)	
Operative time					
< 280 min	23	(79%)	6	(21%)	0.976
≥ 280 min	15	(79%)	4	(21%)	
Estimated blood loss					
< 50 mL	32	(82%)	7	(18%)	0.327
≥50 mL	6	(67%)	3	(33%)	
Intraoperative blood transfusion					
No	37	(79%)	10	(21%)	0.492
Yes	1	(100%)	0	(0%)	

^a^Univariate analyses included Chi squared and Fisher’s exact probability tests.

**Table 3:** The profile of postoperative liver dysfunction

	**Postoperative liver dysfunction**							**Univariate** ^a^
**Variables**	**All patient**	(*n* = **48)**	**Grade2**	(***n*** =** 38)**	**Grade3**	(***n*** =** 10)**		***p***-**value**
Postoperative days of onset							0.196	
Median (range)	7	(1–12)	7	(5–10)	6	(1–12)		
Peak AST							0.214	
Median (range)	94	(15–347)	91	(15–140)	140	(30–347)		
Peak ALT							0.118	
Median (range)	90	(14–342)	87	(14–165)	145	(14–342)		
Peak ALP							**0.026**	
Median (range)	221	(94–792)	202	(94–792)	307	(125–653)		
Peak γ-GTP							0.065	
Median (range)	61	(11–606)	56	(11–456)	86	(39–606)		
Peak T-Bil							0.319	
Median (range)	0.61	(0.33–5.14)	0.59	(0.33–2.46)	0.67	(0.48–5.14)		

*AST* Aspartate transaminase, *ALT* Alanine aminotransferase, *ALP* Alkaline phosphatase

*GGT* Gamma-glutamyl transpeptidase, *T-Bil* Total bilirubin

^a^Univariate analysis included Chi squared and Fisher’s exact probability tests

**Table 4:** Disease-free survival after surgery for Stage III colorectal cancer

	Variable		Univariate^a^	Multivariate^b^		
		*n* = 249	*p*-value	HR ^c^	95% CI^d^	*p*-value
Gender	male vs female	145 vs 104	0.904			
Age (years)	≥ 65 vs < 65	165 vs 84	0.762			
T stage (TNM classification)	T4 vs T1 ~ 3	39 vs 210	**0.025**	2.09	1.123–3.692	**0.022**
N stage (TNM classification)	N2, 3 vs N1	81 vs 168	0.254			
Lymphatic invasion	present vs absent	159 vs 90	0.485			
Venous invasion	present vs absent	156 vs 93	**0.043**	2.13	1.013–3.117	**0.041**
Adjuvant chemotherapy	Yes vs No	171 vs 78	0.151			
Anastomotic leakage	present vs absent	6 vs 243	**0.019**	2.65	0.632–7.512	0.158
Postoperative liver dysfunction	Yes vs No	44 vs 205	**0.001**	2.75	1.544–4.728	**0.001**

^a^Kaplan-Meier method; significance was determined by log-rank test

^b^Multivariate survival analysis was performed using Cox's proportional hazard model

^c^ *HR* Hazard ratio

^d^ *CI* Confidence interval

### Fatty liver assessment

To assess the impact of fatty liver on liver dysfunction, we used preoperative CT images to calculate the liver-to-spleen ratio (L/S ratio) [9, 10]. The Hounsfield unit (HU) values of four regions of interest (ROIs), two in the liver (one from each lobe) and two in the spleen, were measured in the preoperative plain CT of each patient. All ROIs were a circle of 2-cm diameter, avoiding heterogeneous areas such as blood vessels and cysts. The L/S ratio was calculated as the quotient of the average HU value from both ROIs in the liver and spleen.

### Statistical analysis

Statistical analysis was performed using JMP version 10 (ASA Institute, Cary, NC). To compare the clinicopathological characteristics of the two groups, Chi-squared and Fisher’s exact probability tests were performed for categorical variables, and Student’s t-tests and Mann–Whitney *U*-tests were performed with unpaired continuous data. Survival curves were estimated using the Kaplan–Meier method, and statistical differences were examined using the log-rank test. Univariate and multivariate survival analyses were performed using the likelihood ratio test of the stratified Cox proportional hazards model. *P* < 0.05 was considered statistically significant.

## Results

### Clinicopathological factors of postoperative liver dysfunction

A total of 48 patients had postoperative liver dysfunction (Grade 2: *n* = 38; Grade 3: *n* = 10). By univariate analysis, postoperative liver dysfunction was significantly associated with the use of low-molecular-weight heparin (*P* = 0.031), and a low liver-to-spleen ratio on preoperative plain CT (L/S ratio < 1.268; *P* = 0.001; Table 1). Multivariate analysis also revealed that the L/S ratio (*P* = 0.002, HR 2.66 [95% CI: 1.42–5.18]) was an independent risk factor for postoperative liver dysfunction (Table 1**)**. Comorbidities, such as chronic liver disease, were not significantly associated with postoperative liver dysfunction (Supplementary Table 2).

### Risk factors influencing the severity of postoperative liver dysfunction

Ten patients (20.8% (10/48)) were classified with severe postoperative liver dysfunction, with the remaining 38 (79.2% (38/48)) patients classified as grade 2. Univariate analysis revealed that severe postoperative liver dysfunction was significantly associated with lower albumin-bilirubin scores (ALBI ≥ -2.6; *P* = 0.032). There were no correlations between the severity of postoperative liver dysfunction and gender, age, obesity, drinking habits, use of low-molecular-weight heparin, neoadjuvant chemotherapy, L/S ratio, FIB-4 index, Hepatic stenosis index, surgical approach, tumor location, anastomotic leakage, surgical site infection, postoperative ileus, operative time, blood loss, or intraoperative blood transfusion (Table 2).

### Hepatobiliary enzyme abnormalities

Hepatobiliary enzyme abnormalities are seen in various types of liver disease, including hepatitis virus infection, alcohol-induced liver injury, drug-induced liver injury, nonalcoholic fatty liver disease, cirrhosis, and hepatocellular carcinoma. The pathophysiology of liver dysfunction is classified into three types: hepatocellular, cholestasis, and mixed [11]. However, the pattern of hepatobiliary enzyme abnormalities after surgery for CRC remains unknown. Therefore, we assessed liver dysfunction profiles according to the type of elevated enzyme (Supplementary Table 3). There was no significant difference in the median interval between surgery and the onset of liver dysfunction between the severity groups (Table 2). Regarding the peak enzyme values, ALP was significantly higher in patients with severe postoperative liver dysfunction (*P* = 0.026; Table 2).

### Prognostic impact of postoperative liver dysfunction in Stage III CRC

Figure 2 shows the overall survival curves and disease-free survival (DFS) curves of patients with and without postoperative liver dysfunction following radical resection for Stage III colorectal cancer. Patients with postoperative liver dysfunction showed a significantly poorer prognosis than patients without liver dysfunction (*P* = 0.0009). Univariate and multivariate analyses using Cox’s proportional hazards model revealed that postoperative liver dysfunction was an independent prognostic factor affecting DFS (*P* = 0.001, HR 2.50, 95% CI: 1.20–4.91; Table 3).

## Discussion

Our study reveals that postoperative liver dysfunction is strongly associated with worse DFS after radical surgery for Stage III colorectal cancer. Furthermore, a low L/S ratio on preoperative CT was strongly associated with postoperative liver dysfunction in patients with colorectal cancer. Postoperative complications increase tumor recurrence rate and reduce overall survival in patients with colorectal cancer [5, 12]. However, to our knowledge, this is the first study to demonstrate that postoperative liver dysfunction independently contributes to a poorer prognosis among colorectal cancer patients.

To clarify the mechanism by which postoperative liver dysfunction affects long-term prognosis, we investigated whether liver metastasis is present at the surgery for CRC and whether the presence of postoperative liver dysfunction influences adjuvant chemotherapy. Regarding the potential of liver metastases, fatty liver, which causes postoperative liver dysfunction, makes it difficult to detect liver metastasis [13]. Since a fatty liver is an independent risk factor for postoperative liver dysfunction, its recurrence form was also examined. However, there was no significant difference in the frequency of liver metastasis recurrence between patients with or without postoperative liver dysfunction. Postoperative complications are significantly associated with worse rates and delays in adjuvant chemotherapy [14, 15]. However, in this study, the induction rate of postoperative adjuvant chemotherapy did not differ between patients with or without postoperative liver dysfunction (Supplementary Table 4). Several previous reports describe an association between preoperative liver dysfunction and poor prognosis [16, 17]. The serum AST/ALT ratio (a biomarker for cirrhosis), insulin resistance, and alcoholic liver disease are valid prognostic markers for DFS in Stage II and III CRC patients [16]. In addition, non-alcoholic fatty liver disease (NAFLD) and non-alcoholic steatohepatitis (NASH), caused by the accumulation of excess fat in hepatocytes, are also prognostic factors for tumor recurrence after radical surgical resection [17–19]. These reports suggest that chronic inflammation of the liver affects CRC prognosis. In this study, we found an association between postoperative liver dysfunction and the L/S ratio. The absence of a direct prognostic correlation between multiple liver reserve indicators, namely L/S ratio, ALBI score, FIB-4 index, and Hepatic stenosis index, and CRC implies that the unfavorable prognosis of CRC could potentially be attributed to chronic inflammation resulting from fatty liver (Supplementary Fig. 1).

Notably, our study revealed that a fatty liver, closely associated with lifestyle factors such as diet and exercise, increases the risk of preoperative liver dysfunction and may be a modifiable risk factor for perioperative liver dysfunction. Reportedly, a six-month intervention to modify the lifestyle of patients with NAFLD reduced their body weight, improving markers of fatty liver and liver function [20]. Moreover, sodium-glucose cotransporter 2 inhibitors improve the fatty liver in patients with NAFLD and type 2 diabetes mellitus [21]. As demonstrated in previous interventions aimed at modifying lifestyle factors, improvement in markers of fatty liver and liver function in patients with non-alcoholic fatty liver disease (NAFLD) can be achieved. These findings suggest that lifestyle improvements and appropriate preoperative interventions may prevent postoperative liver dysfunction and ultimately improve the prognosis for CRC patients, particularly those with a fatty liver. Therefore, it is essential to consider lifestyle modifications and preoperative interventions to mitigate perioperative liver dysfunction and ultimately improve the prognosis for CRC patients.

There were several limitations of this study, including its retrospective nature and single-center sample. Large prospective cohort studies are needed to validate these findings before clinical application. Additionally, this study included patients with colorectal cancer in various locations and stages. Therefore, several perioperative factors might have also influenced postoperative liver dysfunction. However, it was difficult to completely remove the influence of perioperative factors on postoperative liver status.

## Conclusions

Our study found that postoperative liver dysfunction might be a crucial mechanism leading to poor prognosis in patients with CRC. Postoperative liver dysfunction was associated with poor long-term outcomes in patients with Stage III colorectal cancer. The occurrence of postoperative liver dysfunction in colorectal cancer is significantly associated with a low liver-to-spleen ratio on preoperative plain CT. Preoperative interventions for patients with the potential for a fatty liver may improve the prognosis for CRC.

## Supplementary Information

**Additional file 1: Figure S1. **Hepatic reserve index and prognostic evaluation

**Additional file 2: Table S1. **Definition of Hepatobiliary Enzyme Abnormalities inCommon Terminology Criteria for Adverse Events version 5.0.

**Additional file 3: Table S2. **The association between postoperative comorbiditiesand postoperative liver dysfunction.

**Additional file 4: Table S3. **Changes in hepatobiliary enzymes in patient withpostoperative liver dysfunction.

**Additional file 5: Table S4. **The association between postoperative liverdysfunction and Stage III CRC patient characteristics.

## Data Availability

All data generated or analyzed during this study are included in this article. Further enquiries can be directed to the corresponding author.

## Abbreviations

CTCAE: Common Terminology Criteria for Adverse Events
CRC: Colorectal cancer
CT: Computed tomography
L/S: Liver-to-spleen
AST: Aspartate transaminase
ALT: Alanine aminotransferase
HU: Hounsfield unit
ROIs: Regions of interest
DFS: Disease-free survival
NAFLD: Non-alcoholic fatty liver disease
