# Supplementary material for: Postoperative liver dysfunction is associated with poor long-term outcomes in patients with colorectal cancer: a retrospective cohort study
Source: BMC Gastroenterol. 2023 Apr 18;23:128. doi: 10.1186/s12876-023-02762-y (PMC10114433; doi:10.1186/s12876-023-02762-y)
Supplement: Supplementary file 1 — Additional file 1: Figure S1. Hepatic reserve index and prognostic evaluation [file 12876_2023_2762_MOESM1_ESM.pptx]

## Slide 1
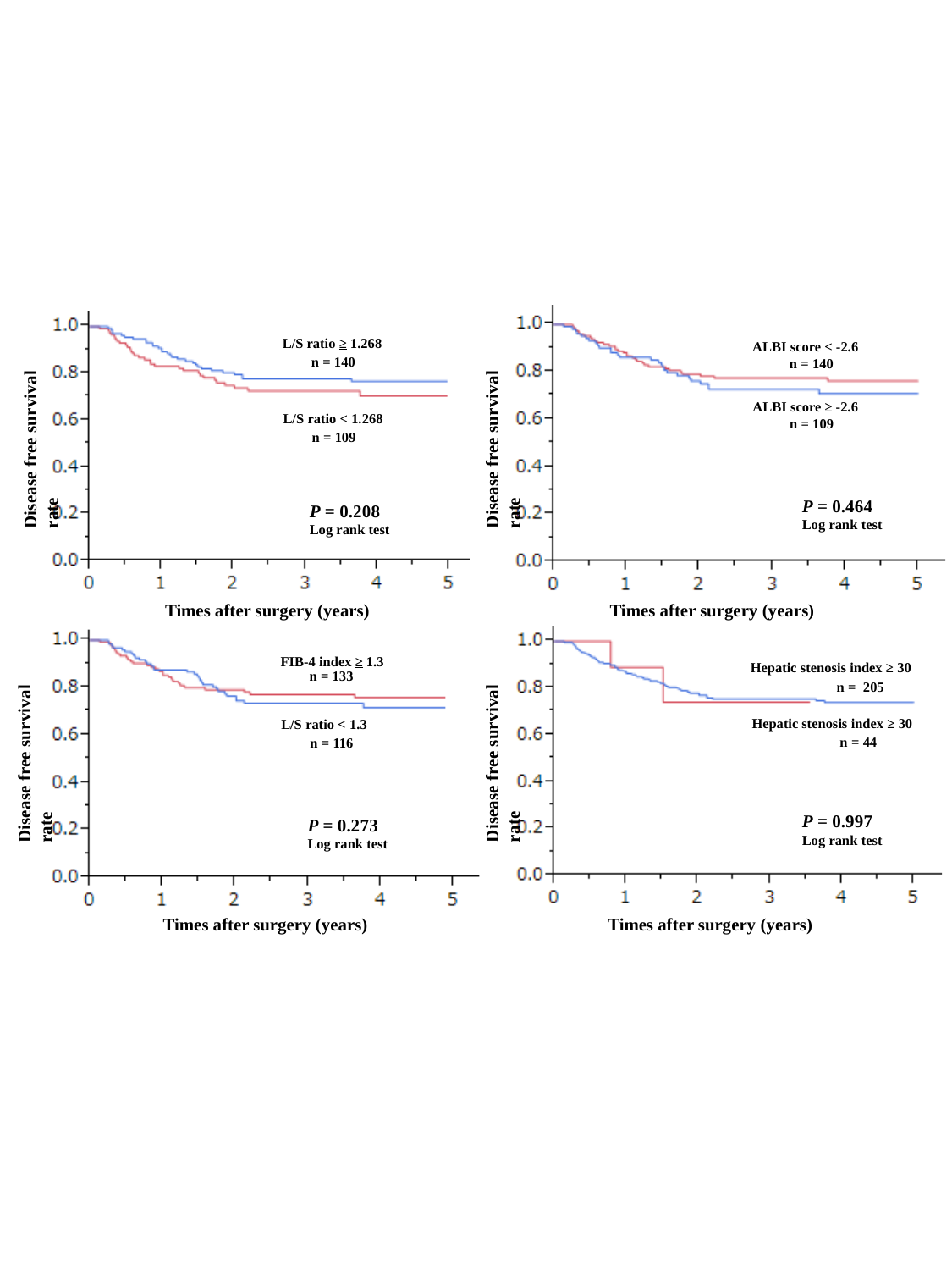

Disease free survival rate
L/S ratio ≥ 1.268
ALBI score < -2.6
Disease free survival rate
n = 140
n = 140
ALBI score ≥ -2.6
L/S ratio < 1.268
n = 109
n = 109
P = 0.464
Log rank test
P = 0.208
Log rank test
Times after surgery (years)
Times after surgery (years)
FIB-4 index ≥ 1.3
Disease free survival rate
Disease free survival rate
Hepatic stenosis index ≥ 30
n = 133
n = 205
Hepatic stenosis index ≥ 30
L/S ratio < 1.3
n = 44
n = 116
P = 0.997
Log rank test
P = 0.273
Log rank test
Times after surgery (years)
Times after surgery (years)
