# Supplementary material for: Postoperative liver dysfunction is associated with poor long-term outcomes in patients with colorectal cancer: a retrospective cohort study
Source: BMC Gastroenterol. 2023 Apr 18;23:128. doi: 10.1186/s12876-023-02762-y (PMC10114433; doi:10.1186/s12876-023-02762-y)
Supplement: Supplementary file 2 — Additional file 2: Table S1. Definition of Hepatobiliary Enzyme Abnormalities inCommon Terminology Criteria for Adverse Events version 5.0. [file 12876_2023_2762_MOESM2_ESM.docx]

**Table S1.** Definition of Hepatobiliary Enzyme Abnormalities in Common Terminology Criteria for Adverse Events version 5.0

| **MedDRA Code** | **MedDRA SOC** | **CTCAE Term** | **Grade 1** | **Grade 2** |
| --- | --- | --- | --- | --- |
| 10001551 | Investigations | Alanine aminotransferase increased | >ULN - 3.0 x ULN if baseline was normal;  1.5 - 3.0 x baseline if baseline was abnormal | >3.0 - 5.0 x ULN if baseline was normal;  >3.0 - 5.0 x baseline if baseline was abnormal |
| 10001675 | Investigations | Alkaline phosphatase increased | >ULN - 2.5 x ULN if baseline was normal;  2.0 - 2.5 x baseline if baseline was abnormal | >2.5 - 5.0 x ULN if baseline was normal; >2.5 - 5.0 x baseline if baseline was abnormal |
| 10003481 | Investigations | Aspartate aminotransferase increased | >ULN - 3.0 x ULN if baseline was normal;  1.5 - 3.0 x baseline if baseline was abnormal | >3.0 - 5.0 x ULN if baseline was normal;  >3.0 - 5.0 x baseline if baseline was abnormal |
| 10005364 | Investigations | Blood bilirubin increased | >ULN - 1.5 x ULN if baseline was normal;  >1.0 - 1.5 x baseline if baseline was abnormal | >1.5 - 3.0 x ULN if baseline was normal; >1.5 - 3.0 x baseline if baseline was abnormal |
|  |  |  |  |  |
| **MedDRA Code** | **MedDRA SOC** | **CTCAE Term** | **Grade 3** | **Grade 4** |
| 10001551 | Investigations | Alanine aminotransferase increased | >5.0 - 20.0 x ULN if baseline was normal;  >5.0 - 20.0 x baseline if baseline was abnormal | >20.0 x ULN if baseline was normal;  >20.0 x baseline if baseline was abnormal |
| 10001675 | Investigations | Alkaline phosphatase increased | >5.0 - 20.0 x ULN if baseline was normal;  >5.0 - 20.0 x baseline if baseline was abnormal | >20.0 x ULN if baseline was normal;  >20.0 x baseline if baseline was abnormal |
| 10003481 | Investigations | Aspartate aminotransferase increased | >5.0 - 20.0 x ULN if baseline was normal;  >5.0 - 20.0 x baseline if baseline was abnormal | >20.0 x ULN if baseline was normal;  >20.0 x baseline if baseline was abnormal |
| 10005364 | Investigations | Blood bilirubin increased | >3.0 - 10.0 x ULN if baseline was normal;  >3.0 - 10.0 x baseline if baseline was abnormal | >10.0 x ULN if baseline was normal;  >10.0 x baseline if baseline was abnormal |
|  |  |  |  |  |
| **MedDRA Code** | **MedDRA SOC** | **CTCAE Term** | **Definition** | |
| 10001551 | Investigations | Alanine aminotransferase increased | A finding based on laboratory test results that indicate an increase in the level of alanine aminotransferase (ALT or SGPT) in the blood specimen. | |
| 10001675 | Investigations | Alkaline phosphatase increased | A finding based on laboratory test results that indicate an increase in the level of alkaline phosphatase in a blood specimen. | |
| 10003481 | Investigations | Aspartate aminotransferase increased | A finding based on laboratory test results that indicate an increase in the level of aspartate aminotransferase (AST or SGOT) in a blood specimen. | |
| 10005364 | Investigations | Blood bilirubin increased | A finding based on laboratory test results that indicate an abnormally high level of bilirubin in the blood.  Excess bilirubin is associated with jaundice. | |
