# Supplementary material for: Postoperative liver dysfunction is associated with poor long-term outcomes in patients with colorectal cancer: a retrospective cohort study
Source: BMC Gastroenterol. 2023 Apr 18;23:128. doi: 10.1186/s12876-023-02762-y (PMC10114433; doi:10.1186/s12876-023-02762-y)
Supplement: Supplementary file 3 — Additional file 3: Table S2. The association between postoperative comorbiditiesand postoperative liver dysfunction. [file 12876_2023_2762_MOESM3_ESM.docx]

**Table S2.** The association between postoperative comorbidities and postoperative liver dysfunction

|  |  |  |  |  |  |  |  |  |
| --- | --- | --- | --- | --- | --- | --- | --- | --- |
|  |  |  | **Postoperative liver dysfunction** | | | | **Univariate ^a^** |  |
|  | **Variables** | | **(+)** | **(n=48)** | **(-)** | **(n=312)** | **p-value** |  |
|  | Hypertension | |  |  |  |  |  |  |
|  |  | No | 35 | (15%) | 197 | (85%) | 0.179 |  |
|  |  | Yes | 13 | (10%) | 115 | (90%) |  |  |
|  | Hyperlipidemia | |  |  |  |  |  |  |
|  |  | No | 44 | (14%) | 276 | (86%) | 0.496 |  |
|  |  | Yes | 4 | (10%) | 36 | (90%) |  |  |
|  | Cardiovascular disease | |  |  |  |  |  |  |
|  |  | No | 44 | (14%) | 270 | (86%) | 0.298 |  |
|  |  | Yes | 4 | (9%) | 42 | (91%) |  |  |
|  | Diabetes mellitus | |  |  |  |  |  |  |
|  |  | No | 44 | (14%) | 263 | (86%) | 0.153 |  |
|  |  | Yes | 4 | (8%) | 49 | (92%) |  |  |
|  | Cerebral disease | |  |  |  |  |  |  |
|  |  | No | 45 | (13%) | 299 | (87%) | 0.534 |  |
|  |  | Yes | 3 | (19%) | 13 | (81%) |  |  |
|  | Respiratory disease | |  |  |  |  |  |  |
|  |  | No | 48 | (14%) | 300 | (86%) | 0.062 |  |
|  |  | Yes | 0 | (0%) | 12 | (100%) |  |  |
|  | Endocrine disease | |  |  |  |  |  |  |
|  |  | No | 42 | (12%) | 296 | (88%) | 0.073 |  |
|  |  | Yes | 6 | (27%) | 16 | (73%) |  |  |
|  | Autoimmune disease | |  |  |  |  |  |  |
|  |  | No | 47 | (14%) | 299 | (86%) | 0.453 |  |
|  |  | Yes | 1 | (7%) | 13 | (93%) |  |  |
|  |  |  |  |  |  |  |  |  |
|  | ^a^ Univariate analysis included Chi squared and Fisher’s exact probability tests. | | | | | | | |
