# Supplementary material for: Postoperative liver dysfunction is associated with poor long-term outcomes in patients with colorectal cancer: a retrospective cohort study
Source: BMC Gastroenterol. 2023 Apr 18;23:128. doi: 10.1186/s12876-023-02762-y (PMC10114433; doi:10.1186/s12876-023-02762-y)
Supplement: Supplementary file 4 — Additional file 4: Table S3. Changes in hepatobiliary enzymes in patient withpostoperative liver dysfunction. [file 12876_2023_2762_MOESM4_ESM.docx]

**Table S3.** Changes in hepatobiliary enzymes in patient with postoperative liver dysfunction

|  |  |  | **Postoperative liver dysfunction** | | | | **Univariate ^a^** |  |
| --- | --- | --- | --- | --- | --- | --- | --- | --- |
|  | **Variables** | | **(+)** | **(n=48)** | **(-)** | **(n=312)** | **p-value** |  |
|  | AST (Median (range)) |  |  |  |  |  |  |  |
|  | preoperative |  | 20 | (11-28) | 19 | (10-30) | 0.074 |  |
|  | POD1 |  | 26 | (11-152) | 20 | (11-45) | **0.032** |  |
|  | POD3 |  | 38 | (13-128) | 22 | (9-47) | **<0.001** |  |
|  | POD5 |  | 47 | (12-347) | 22 | (8-89) | **<0.001** |  |
|  | POD7 |  | 140 | (15-224) | 32 | (8-85) | **<0.001** |  |
|  | ALT (Median (range)) |  |  |  |  |  |  |  |
|  | preoperative |  | 16 | (8-29) | 13 | (3-36) | 0.04 |  |
|  | POD1 |  | 22 | (9-114) | 16 | (3-40) | **0.022** |  |
|  | POD3 |  | 30 | (11-109) | 16 | (3-58) | **<0.001** |  |
|  | POD5 |  | 33 | (11-187) | 14 | (3-80) | **<0.001** |  |
|  | POD7 |  | 83 | (14-342) | 25 | (3-77) | **<0.001** |  |
|  | ALP (Median (range)) |  |  |  |  |  |  |  |
|  | preoperative |  | 198 | (74-301) | 212 | (52-312) | 0.333 |  |
|  | POD1 |  | 188 | (68-452) | 194 | (54-282) | 0.477 |  |
|  | POD3 |  | 201 | (66-573) | 188 | (62-406) | 0.386 |  |
|  | POD5 |  | 187 | (66-552) | 162 | (74-477) | 0.208 |  |
|  | POD7 |  | 209 | (94-653) | 172 | (69-570) | **0.016** |  |
|  | γ-GTP (Median (range)) |  |  |  |  |  |  |  |
|  | preoperative |  | 21 | (11-72) | 19 | (11-42) | 0.256 |  |
|  | POD1 |  | 25 | (11-128) | 20 | (10-68) | **0.037** |  |
|  | POD3 |  | 34 | (9-153) | 23 | (9-101) | **0.005** |  |
|  | POD5 |  | 38 | (9-205) | 21 | (7-128) | **<0.001** |  |
|  | POD7 |  | 59 | (11-606) | 25 | (8-242) | **<0.001** |  |
|  | T-Bil (Median (range)) |  |  |  |  |  |  |  |
|  | preoperative |  | 0.72 | (0.40-1.03) | 0.63 | (0.40-1.20) | 0.058 |  |
|  | POD1 |  | 0.64 | (0.33-3.13) | 0.56 | (0.35-1.33) | 0.103 |  |
|  | POD3 |  | 0.78 | (0.48-5.14) | 0.58 | (0.28-1.44) | **0.039** |  |
|  | POD5 |  | 0.63 | (0.37-2.46) | 0.60 | (0.23-1.56) | 0.342 |  |
|  | POD7 |  | 0.67 | (0.48-2.46) | 0.54 | (0.21-1.26) | **0.034** |  |
|  |  |  |  |  |  |  |  |  |
|  | *POD* postoperative day |  |  |  |  |  |  |  |
|  | ^a^ Univariate analysis included Chi squared and Fisher’s exact probability tests. | | | | | | | |
