# Supplementary material for: Postoperative liver dysfunction is associated with poor long-term outcomes in patients with colorectal cancer: a retrospective cohort study
Source: BMC Gastroenterol. 2023 Apr 18;23:128. doi: 10.1186/s12876-023-02762-y (PMC10114433; doi:10.1186/s12876-023-02762-y)
Supplement: Supplementary file 5 — Additional file 5: Table S4. The association between postoperative liverdysfunction and Stage III CRC patient characteristics. [file 12876_2023_2762_MOESM5_ESM.docx]

**Table S4.** The association between postoperative liver dysfunction and Stage III CRC patient characteristics

|  |  |  | **Postoperative liver dysfunction** | | | | **Univariate ^a^** |  |
| --- | --- | --- | --- | --- | --- | --- | --- | --- |
|  | **Variables** | | **(+)** | **(n=44)** | **(-)** | **(n=205)** | **p-value** |  |
|  | Gender | |  |  |  |  |  |  |
|  |  | Female | 18 | (18%) | 86 | (82%) | 0.898 |  |
|  |  | Male | 26 | (17%) | 119 | (83%) |  |  |
|  | Age (years) | |  |  |  |  |  |  |
|  |  | < 65 | 19 | (21%) | 65 | (79%) | 0.150 |  |
|  |  | ≥ 65 | 25 | (14%) | 140 | (86%) |  |  |
|  | Body composition | |  |  |  |  |  |  |
|  |  | < BMI 25 | 35 | (18%) | 165 | (82%) | 0.887 |  |
|  |  | ≥ BMI 25 | 9 | (18%) | 40 | (82%) |  |  |
|  | Drinking | |  |  |  |  |  |  |
|  |  | No | 18 | (15%) | 105 | (85%) | 0.213 |  |
|  |  | Yes | 26 | (21%) | 100 | (79%) |  |  |
|  | Neoadjuvant chemotherapy | |  |  |  |  |  |  |
|  |  | No | 36 | (18%) | 167 | (82%) | 0.729 |  |
|  |  | Yes | 8 | (17%) | 38 | (83%) |  |  |
|  | L/S ratio | |  |  |  |  |  |  |
|  |  | > 1.268 | 14 | (10%) | 126 | (90%) | **0.003** |  |
|  |  | < 1.268 | 30 | (28%) | 79 | (72%) |  |  |
|  | ALBI score | |  |  |  |  |  |  |
|  |  | < -2.6 | 29 | (21%) | 111 | (79%) | 0.149 |  |
|  |  | > -2.6 | 15 | (14%) | 94 | (86%) |  |  |
|  | FIB-4 index | |  |  |  |  |  |  |
|  |  | < 1.3 | 20 | (17%) | 96 | (83%) | 0.864 |  |
|  |  | > 1.3 | 24 | (18%) | 109 | (82%) |  |  |
|  | Hepatic stenosis index | |  |  |  |  |  |  |
|  |  | < 30 | 42 | (18%) | 163 | (82%) | 0.925 |  |
|  |  | > 30 | 2 | (17%) | 42 | (83%) |  |  |
|  | Surgical approach | |  |  |  |  |  |  |
|  |  | Laparotomy | 3 | (14%) | 18 | (86%) | 0.663 |  |
|  |  | Laparoscopic | 41 | (18%) | 187 | (82%) |  |  |
|  | Tumor location | |  |  |  |  |  |  |
|  |  | colon | 26 | (17%) | 123 | (83%) | 0.911 |  |
|  |  | rectum | 18 | (18%) | 82 | (82%) |  |  |
|  | Operative time | |  |  |  |  |  |  |
|  |  | < 280 min | 28 | (16%) | 145 | (84%) | 0.360 |  |
|  |  | ≥ 280 min | 16 | (21%) | 60 | (79%) |  |  |
|  | Estimated blood loss | |  |  |  |  |  |  |
|  |  | < 50 mL | 38 | (19%) | 164 | (81%) | 0.312 |  |
|  |  | 50 mL < | 6 | (13%) | 41 | (87%) |  |  |
|  | Intraoperative blood transfusion | |  |  |  |  |  |  |
|  |  | No | 43 | (18%) | 198 | (82%) | 0.684 |  |
|  |  | Yes | 1 | (13%) | 7 | (87%) |  |  |
|  | Surgical site infection | |  |  |  |  |  |  |
|  |  | No | 38 | (18%) | 170 | (82%) | 0.783 |  |
|  |  | Yes | 6 | (15%) | 35 | (85%) |  |  |
|  | Postoperative ileus | |  |  |  |  |  |  |
|  |  | No | 43 | (18%) | 197 | (82%) | 0.479 |  |
|  |  | Yes | 1 | (11%) | 8 | (89%) |  |  |
|  | Anastomotic leakage | |  |  |  |  |  |  |
|  |  | No | 41 | (17%) | 202 | (83%) | 0.082 |  |
|  |  | Yes | 3 | (50%) | 3 | (50%) |  |  |
|  | Adjuvant chemotherapy | |  |  |  |  |  |  |
|  |  | No | 12 | (15%) | 66 | (85%) | 0.518 |  |
|  |  | Yes | 32 | (19%) | 139 | (81%) |  |  |
|  | Liver metastasis | |  |  |  |  |  |  |
|  |  | No | 37 | (16%) | 191 | (84%) | 0.069 |  |
|  |  | Yes | 7 | (33%) | 14 | (67%) |  |  |
|  |  |  |  |  |  |  |  |  |
|  | ^a^ Univariate analysis included Chi squared and Fisher’s exact probability tests. | | | | | | | |
